# Data on anti-corrosion characteristics of eco-friendly inhibitive extract on the hot corrosion degradation trend of A6063 aluminum alloy in 1.0 M HCl solution

**DOI:** 10.1016/j.dib.2018.04.007

**Published:** 2018-04-07

**Authors:** O.S.I. Fayomi, J. Fayomi, E.E. Elemike

**Affiliations:** a Department of Mechanical Engineering, Covenant University, P.M.B. 1023 Ota, Nigeria; b Department of Chemical, Metallurgical and Materials Engineering, Tshwane University of Technology, P.M.B. X680 Pretoria, South Africa; c Department of Chemistry, Federal University of Petroleum Resources Effurum, Nigeria

**Keywords:** Inhibitor efficiency, Temperature, HCl solution, *Lasienthera africanum*

## Abstract

The study of *Lasienthera africanum* as corrosion inhibitor for A6063 grade aluminum alloy in 1.0 M HCl acid solution using weight loss method of corrosion study was investigated at 30 °C and 40 °C to check its degradation trend at high varying temperature application. The results revealed that *L. africanum* in 1.0 M acidic environment decreased the corrosion rate at various concentrations considered. Inhibitor efficiency (IE) as high as 98.7 and 94.9% both at 30 °C and 40 °C respectively, was observed.

**Specifications Table**

**Table:** 

Subject area	*Material science*
More specific subject area	*Corrosion science*
Type of data	*Table, figures*
How data was acquired	The data acquired in this work involved the introduction of already prepared concentrations of the inhibitor into separate beakers maintained at room temperature. A total of seven beakers labelled [1], [2], [3], [4], [5] and (X and Y) were used; with 1, 2, 3, 4, 5 containing 100 ml of the acidic extract solution while X and Y were used as the blank (control) for the experiment. The beakers labelled 1–5 contains different volumes of stock solution of the inhibitor with concentrations of 10 mg/L, 20 mg/L, 40 mg/L, 60 mg/L, 80 mg/L which was made up to 100 ml each into different concentration of the acid 1.0 M HCl.
	Already weighed aluminium alloy coupons were then placed in the test solution. Each coupon was retrieved from the test solutions progressively for 1 h in total of 5 h [1]. After the immersion test, the specimens were carefully dip in water and then properly cleaned to remove loose segments of the film of the corroded samples following by degreasing in ethanol and drying with acetone. The difference in weight of the coupons was again taken as the weight loss.
Data format	Raw, Analysed
Experimental factors	The aluminum alloy coupons were properly weighed before and after immersion into the test solution. The inhibitor was test against temperature of 30 °C and 40 °C at different concentration for the period of 5 h.
Experimental features	The depositions were performed between 60 and 300 min at a temperature of 30 °C and 40 °C. The effect of inhibitor on the properties of aluminum alloy was acquired. The framework of temperature variation condition as it influences the corrosion rate and efficiency was properly observed.
Data source location	Department of Chemistry, Federal University of petroleum Resources, Effurun, Delta State. Department of Chemical, Metallurgical and Materials Engineering, Tshwane University of Technology, Pretoria, South Africa and Mechanical Engineering, Covenant University, Ota Ogun State, Nigeria
Data accessibility	Data are available within this article

**Value of the data**

● The data obtained could be used to check the degradation trend of the typed aluminum alloy in acidic environment at varying temperature in the present of inhibitor.
• The data could be used to check the effect of increase in temperature on the inhibition efficiency of the inhibitor used (*Lasienthera africanum)*
• The results obtained shows that the inhibition potency of the inhibitor decreases with time in the contaminated environment.
• The given data will show author in the field of corrosion science the effect of 1.0 M HCl concentration on aluminium corrosion, with or with out inhibitor.
• The data obtained could be used to check the correlation of temperature variation on the corrosion of aluminum coupon in acidic medium.

## 1 Data

The weight losses with depth of depositions were collected and a unique set of experimental frame work data were generated. The immersion process was performed between 60 and 300 min at a varying temperature of 30 °C and 40 °C [2]. The data acquired from the weight loss measurements of the aluminum coupon is presented in Table 1, Table 2, Table 3, Table 4, Table 5, Table 6, Table 7, Table 8, Table 9, Table 10, Table 11, Table 12, Table 13, Table 14 below. From the weight loss result, the corrosion rates were calculated and the inhibitor efficiency obtained. The data obtained shows that the rate of corrosion decreases with increase in the concentration of inhibitors, this is also true for the varying temperature though with increase in time the inhibitor efficiency decreases [3], [4].

**Table 1:** Aluminum coupon in 1.0 M HCl at 30 °C without *Lasienthera africanum* extract(control 1).

**Time(min)**	**Initial weight of specimen, *W***_**1**_	**Final weight of specimen, *W***_**F**_	**Weight loss, ∆*W***_**(g)**_	**Inhibition efficiency (%)**	**Corrosion rate (mm/yr.)**
60	0.9724	0.9559	0.0165	–	44.61
120	0.9724	0.8343	0.1381	–	186.69
180	0.9724	0.6980	0.2744	–	247.30
240	0.9724	0.6450	0.3274	–	221.30
300	0.9724	0.6346	O.3378	–	182.66

**Table 2:** Aluminum coupon in 1.0 M HCl at 30 °C without *Lasienthera africanum* extract(control 2).

**Time(min)**	**Initial weight of specimen, *W***_**1**_	**Final weight of specimen, *W***_**f**_	**Weight loss, ∆*W***_**(g)**_	**Inhibition efficiency (%)**	**Corrosion rate (mm/yr.)**
60	0.9958	0.9644	0.0314	–	84.90
120	0.9958	0.8249	0.1709	–	231.03
180	0.9958	0.7287	0.2671	–	240.72
240	0.9958	0.6831	0.3119	–	216.82
300	0.9958	0.6641	0.3317	–	179.36

**Table 3:** Aluminum coupon in 1.0 M HCl at 30 °C containing 10 mg/l *Lasienthera africanum* extract.

**Time(min)**	**Initial weight of specimen, *W***_**1**_	**Final weight of specimen, *W***_**f**_	**Weight loss, ∆*W***_**(g)**_	**Inhibition efficiency (%)**	**Corrosion rate (mm/yr.)**
60	1.0211	1.0151	0.0060	74.9	16.22
120	1.0211	0.9750	0.0461	70.2	62.32
180	1.0211	0.8715	0.1496	44.7	134.82
240	1.0211	0.7257	0.2954	7.6	199.67
300	1.0211	0.7087	0.3124	6.7	168.93

**Table 4:** Aluminum coupon in 1.0 M HCl at 30 °C containing 20 mg/l *Lasienthera africanum* extract.

**Time(min)**	**Initial weight of specimen, *W***_**1**_	**Final weight of specimen, *W***_**f**_	**Weight loss, ∆*W***_**(g)**_	**Inhibition efficiency (%)**	**Corrosion rate (mm/yr.)**
60	1.3696	1.3651	0.0045	81.2	12.17
120	1.3696	1.3400	0.0296	80.4	40.01
180	1.3696	1.2654	0.1042	61.5	93.91
240	1.3696	1.1072	0.2625	17.8	177.43
300	1.3696	1.0699	0.2997	10.4	162.06

**Table 5:** Aluminum coupon in 1.0 M HCl at 30 °C containing 40 mg/l *Lasienthera africanum* extract.

**Time(min)**	**Initial weight of specimen, *W***_**1**_	**Final weight of specimen, *W***_**f**_	**Weight loss, ∆*W***_**(g)**_	**Inhibition efficiency (%)**	**Corrosion rate (mm/yr.)**
60	0.9991	0.9961	0.0030	87.5	8.11
120	0.9991	0.9782	0.0209	86.4	28.25
180	0.9991	0.9384	0.0607	77.5	54.70
240	0.9991	0.7707	0.2284	28.6	154.38
300	0.9991	0.7076	0.2955	12.9	158.79

**Table 6:** Aluminum coupon in 1.0 m HCl at 30 °C containing 60 mg/l *Lasienthera africanum* extract.

**Time(Min)**	**Initial weight of specimen, *W***_**1**_	**Final weight of specimen, *W***_**f**_	**Weight loss, ∆*W***_**(g)**_	**Inhibition efficiency (%)**	**Corrosion rate (mm/yr.)**
60	1.0775	1.0762	0.0013	94.6	3.51
120	1.0775	1.0680	0.0095	93.9	64.80
180	1.0775	1.0393	0.0607	85.9	54.70
240	1.0775	0.8931	0.1844	42.3	124.64
300	1.0775	0.7959	0.2816	15.9	152.27

**Table 7:** Aluminum coupon in 1.0 M HCl at 30 °C containing 80 mg/l *Lasienthera africanum* extract.

**Time(min)**	**Initial weight of specimen, *W***_**1**_	**Final weight of specimen, *W***_**f**_	**Weight loss, ∆*W***_**(g)**_	**Inhibition efficiency (%)**	**Corrosion rate (mm/yr.)**
60	0.9851	0.9848	0.0003	98.7	0.811
120	0.9851	0.9798	0.0053	96.6	7.16
180	0.9851	0.9723	0.0128	95.3	11.54
240	0.9851	0.9197	0.0654	79.5	44.21
300	0.9851	0.8140	0.1711	48.9	92.52

**Table 8:** Aluminum coupon in 1.0 M HCl at 40 °C without *Lasienthera africanum* extract (control 1).

**Time(Min)**	**Initial weight of specimen, *W***_**1**_	**Final weight of specimen, *W***_**f**_	**Weight loss, ∆*W***_**(g)**_	**Inhibition efficiency (%)**	**Corrosion rate (mm/yr.)**
60	1.0247	0.7822	0.2425	–	655.65
120	1.0247	0.6438	0.3809	–	514.92
180	1.0247	0.6077	0.4170	–	375.81
240	1.0247	0.5828	0.4419	–	298.69
300	1.0247	0.5699	O.4548	–	245.93

**Table 9:** Aluminum coupon in 1.0 M HCl at 40 °C without *Lasienthera africanum* extract (control 2).

**Time(Min)**	**Initial weight of specimen, *W***_**1**_	**Final weight of specimen, *W***_**f**_	**Weight loss, ∆*W***_**(g)**_	**Inhibition efficiency (%)**	**Corrosion rate (mm/yr.)**
60	1.0501	0.8224	0.2277	–	615.63
120	1.0501	0.7141	0.3360	–	454.22
180	1.0501	0.6381	0.4120	–	371.31
240	1.0501	0.6171	0.4330	–	292.68
300	1.0501	0.6070	0.4431	–	239.60

**Table 10:** Aluminum coupon in 1.0 M HCl at 40 °C containing 10 mg/l *Lasienthera africanum* extract.

**Time(Min)**	**Initial weight of specimen, *W***_**1**_	**Final weight of specimen, *W***_**f**_	**Weight loss, ∆*W***_**(g)**_	**Inhibition efficiency (%)**	**Corrosion rate (mm/yr.)**
60	1.0044	0.9279	0.0765	67.5	206.83
120	1.0044	0.6844	0.3200	10.7	432.59
180	1.0044	0.6172	0.3872	6.6	348.96
240	1.0044	0.5932	0.4112	6.0	277.94
300	1.0044	0.5781	0.4263	5.1	230.52

**Table 11:** Aluminum coupon in 1.0 M HCl at 40 °C containing 20 mg/l *Lasienthera africanum* extract.

**Time(min)**	**Initial weight of specimen, *W***_**1**_	**Final weight of specimen, *W***_**f**_	**Weight loss, ∆*W***_**(g)**_	**Inhibition efficiency (%)**	**Corrosion rate (mm/yr.)**
60	1.0141	0.9691	0.0450	80.9	121.67
120	1.0141	0.7169	0.2972	17.1	401.77
180	1.0141	0.6286	0.3855	7.0	347.42
240	1.0141	0.6049	0.4092	6.5	276.59
300	1.0141	0.5893	0.4248	5.4	229.71

**Table 12:** Aluminum coupon in 1.0 M HCl at 40 °C containing 40 mg/l *Lasienthera africanum* extract.

**Time(Min)**	**Initial weight of specimen, *W***_**1**_	**Final weight of specimen, *W***_**f**_	**Weight loss, ∆*W***_**(g)**_	**Inhibition efficiency (%)**	**Corrosion rate (mm/yr.)**
60	0.9964	0.9787	0.0177	92.5	47.86
120	0.9964	0.7250	0.2714	24.3	366.89
180	0.9964	0.6154	0.3810	8.1	343.37
240	0.9964	0.5933	0.4031	7.8	272.47
300	0.9964	0.5744	0.4220	6.0	228.19

**Table 13:** Aluminum coupon in 1.0 m HCl at 40 °C containing 60 mg/l *Lasienthera africanum* extract.

**Time(Min)**	**Initial weight of specimen, *W***_**1**_	**Final weight of specimen, *W***_**f**_	**Weight loss, ∆*W***_**(g)**_	**Inhibition efficiency (%)**	**Corrosion rate (mm/yr.)**
60	0.9898	0.9728	0.0170	92.8	45.96
120	0.9898	0.7251	0.2647	26.2	357.84
180	0.9898	0.6109	0.3789	8.6	341.48
240	0.9898	0.5887	0.4011	8.3	271.11
300	0.9898	0.5752	0.4146	7.7	224.19

**Table 14:** Aluminum coupon in 1.0 M HCl at 40 °C containing 80 mg/l *Lasienthera africanum* extract.

**Time(min)**	**Initial weight of specimen, *W***_**1**_	**Final weight of specimen, *W***_**f**_	**Weight loss, ∆*W***_**(g)**_	**Inhibition efficiency (%)**	**Corrosion rate (mm/yr.)**
60	1.0009	0.9889	0.0120	94.9	32.44
120	1.0009	0.7408	0.2601	27.4	351.62
180	1.0009	0.6215	0.3794	18.5	341.93
240	1.0009	0.6214	0.3876	13.2	261.99
300	1.0009	0.6099	0.3910	12.9	211.43

## 2 Experimental design, materials and methods

The materials employed in this work include Aluminum coupons, Thermometer, Analytical weighing balance, heating mantle, Magnetic stirrer, water bath, desiccator.

### 2.1 Reagents

Hydrochloric acid (HCl), Ethanol, Acetone.

### 2.2 Plant sample

*Lasienthera africanum* extract.

### 2.3 Preparation of aluminum coupons for anti-corrosion study

Aluminum sheets of purity 98.8% were used in this study each sheet was 0.14 cm thick and was mechanically cut into rectangular coupons of dimension 3 cm × 4 cm. The total surface area of the coupon used was 12 cm^2^. These coupons were further polished, degreased in ethanol and dried in acetone. The coupons were then stored in a moisture-free desiccator to avoid contamination before using them for corrosion studies [2]. The initial weight of each sample was taken and recorded. All reagents used were of analytical grade. They were used as sourced with no further purification. An aqueous solution of 1.0 M was used as blank solution.

### 2.4 Preparation of plant extract for corrosion inhibition studies

*L. africanum* leaf sample was bought from Ota market in Ogun state Nigeria and was properly washed. The sample was further dried and ground into fine powder. The acidic leaf extract was prepared by adding 5.0 g of the plant sample in 100 ml 1.0 M HCl [6]. The resulting solution was boiled for 5 h and allowed to stand before filtering. It was observed that 0.2528 g of organic soluble matter dissolved in the acidic medium. This becomes the stock solution and from these concentrations of 10 mg/L, 20 mg/L, 40 mg/L, 60 mg/L, 80 mg/L were made [7].

### 2.5 Weight loss measurement

This work involved the introduction of already prepared concentrations of the inhibitor into separate beakers maintained at room temperature. A total of seven beakers label (1–5 and X and Y) were used; with 1, 2, 3, 4, 5 containing 100 ml of the acidic extract solution while X and Y were used as the blank (control) for the experiment. The beakers label 1–5 contains different volumes of stock solution of the inhibitor with concentrations of 10 mg/L, 20 mg/L, 40 mg/L, 60 mg/L, 80 mg/L which was made up to 100 ml each into different concentration of 1.0 M HCl [8].

**Fig. 1:**
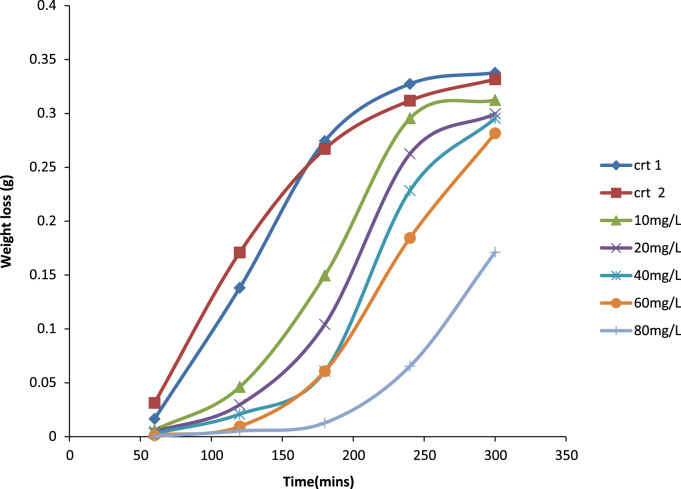
A plot of weight loss against time at 30 °C in 1.0 M HCl.

**Fig. 2:**
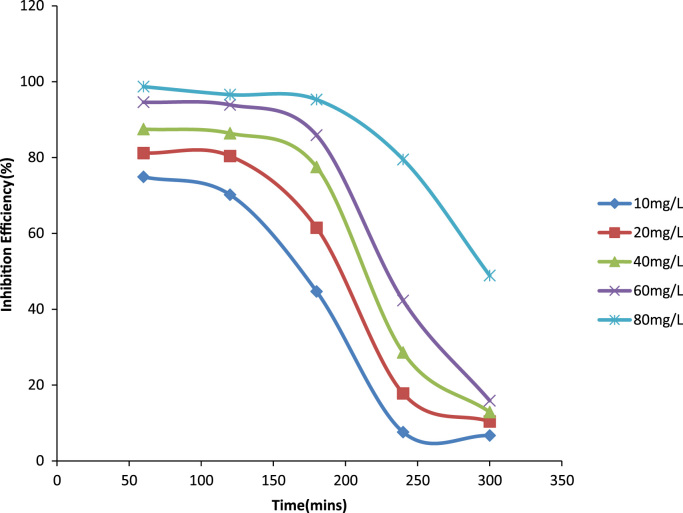
A plot of inhibition efficiency against time at 30 °C IN 1.0 M HCl.

**Fig. 3:**
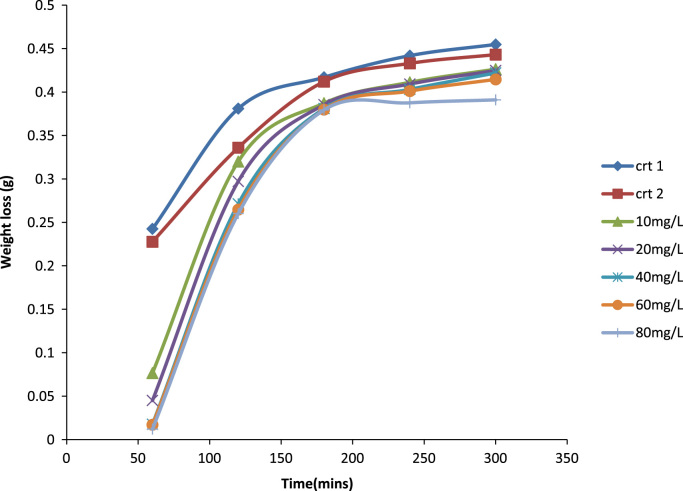
A plot of weight loss against time at 40 °C in 1.0 M HCl.

**Fig. 4:**
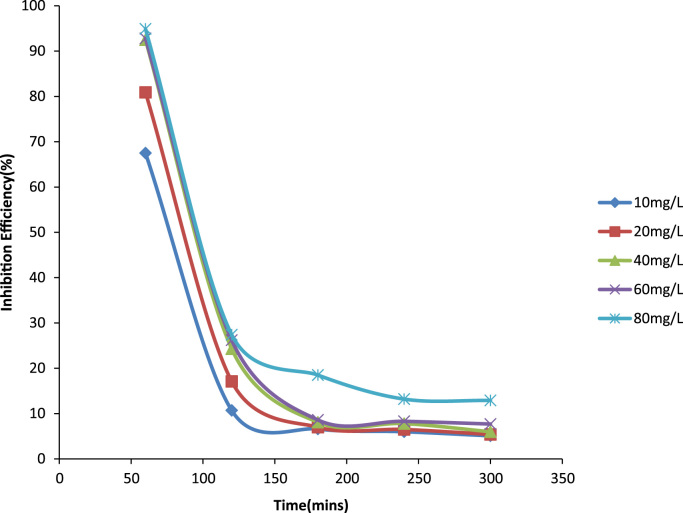
A plot of Inhibition Efficiency against Time at 40 °C in 1.0 M in HCl.

Previously weighed aluminum coupons were then placed in the test solution. Each coupon was retrieved from the test solutions progressively for 1 h in total of 5 h [1]. After the immersion test, the specimens were carefully dip in water and then properly cleaned to remove loose segments of the film of the corroded samples following by degreasing in ethanol and drying with acetone. The difference in weight of the coupons was again taken as the weight loss. From the initial weight of the aluminum coupons, the weight loss, the corrosion rate (CR) and inhibition efficiency were determined [9] (Fig. 1, Fig. 2, Fig. 3, Fig. 4).

## 3 Conclusion

1. The corrosion rates drastically decreased with increase in concentration of the extracts in comparison to the blank. The 80 mg/L extract have the lowest corrosion rate, which depict that the inhibitive action of the extract is concentration dependent.
2. *L. africanum* have been demonstrated to be an effective corrosion inhibitor for 1.0 M HCl environments at 30 and 40 °C
  The corrosion resistance and inhibitor efficiency of aluminium increased with addition of *Lasienthera africanum* as inhibitor. Inhibition efficiency as high as 98.7% and 94.9% in 1.0 M was observed in the varying temperatures.
3. The rate of corrosion of the aluminium in HCl environmental conditions is a function of the concentration of *L. africanum* extract.
