# Supplementary material for: Data on anti-corrosion characteristics of eco-friendly inhibitive extract on the hot corrosion degradation trend of A6063 aluminum alloy in 1.0 M HCl solution
Source: Data Brief. 2018 Apr 7;19:2468–76. doi: 10.1016/j.dib.2018.04.007 (PMC6139366; doi:10.1016/j.dib.2018.04.007)
Supplement: Supplementary file 1 — Supplementary material [file mmc1.docx]

**Conflict of interest**

All the author confirms as no conflict of interest.
